# Protocol for the evaluation of a complex intervention aiming at increased utilisation of primary child health services in Ethiopia: a before and after study in intervention and comparison areas

**DOI:** 10.1186/s12913-020-05151-3

**Published:** 2020-04-21

**Authors:** Della Berhanu, Yemisrach B. Okwaraji, Abebe Bekele Belayneh, Ephrem Tekle Lemango, Nesibu Agonafer, Bizuhan Gelaw Birhanu, Kurabachew Abera, Wuleta Betemariam, Araya Abrha Medhanyie, Muluemebet Abera, Mezgebu Yitayal, Fitsum Woldegebriel Belay, Lars Åke Persson, Joanna Schellenberg

**Affiliations:** 1 Department of Disease Control, London School of Hygiene and Tropical Medicine, Keppel Street, London, WC1E 7HT United Kingdom; 2 Ethiopian Public Health Institute, P.O.Box 1242, Addis Ababa, Ethiopia; 3 International Institute for Primary Health Care-Ethiopia, Addis Ababa, Ethiopia; 4 PATH, Ethiopia Country Program Office, Addis Ababa, Ethiopia; 5 UNICEF, Ethiopia Country Office, Addis Ababa, Ethiopia; 6 Save the Children, Ethiopia Country Office, Addis Ababa, Ethiopia; 7 Last 10 Kilometres, John Snow Institute, Addis Ababa, Ethiopia; 8 School of Public Health, College of Health Sciences, Mekelle University, Mekelle, Ethiopia; 9 Faculty of Public Health, Jimma University, Jimma, Ethiopia; 10 Department of Health Systems and Policy, Institute of Public Health, College of Medicine and Health Sciences, University of Gondar, Gondar, Ethiopia; 11 Hawassa University, College of Medicine and Health Sciences, Hawassa, Ethiopia

**Keywords:** Primary care utilisation, Community-based newborn care, Integrated community case management, Health extension worker, Women’s development army, Community engagement, Quality of care, Ownership, Pragmatic trial, Effectiveness

## Abstract

**Background:**

By expanding primary health care services, Ethiopia has reduced under-five mor4tality. Utilisation of these services is still low, and concerted efforts are needed for continued improvements in newborn and child survival. “Optimizing the Health Extension Program” is a complex intervention based on a logic framework developed from an analysis of barriers to the utilisation of primary child health services. This intervention includes innovative components to engage the community, strengthen the capacity of primary health care workers, and reinforce the local ownership and accountability of the primary child health services. This paper presents a protocol for the process and outcome evaluation, using a pragmatic trial design including before-and-after assessments in both intervention and comparison areas across four Ethiopian regions. The study has an integrated research capacity building initiative, including ten Ph.D. students recruited from Ethiopian Regional Health Bureaus and universities.

**Methods:**

Baseline and endline surveys 2 years apart include household, facility, health worker, and district health office modules in intervention and comparison areas across Amhara, Southern Nations Nationalities and Peoples, Oromia, and Tigray regions. The effectiveness of the intervention on the seeking and receiving of appropriate care will be estimated by difference-in-differences analysis, adjusting for clustering and for relevant confounders. The process evaluation follows the guidelines of the UK Medical Research Council. The implementation is monitored using data that we anticipate will be used to describe the fidelity, reach, dose, contextual factors and cost. The participating Ph.D. students plan to perform in-depth analyses on different topics including equity, referral, newborn care practices, quality-of-care, geographic differences, and other process evaluation components.

**Discussion:**

This protocol describes an evaluation of a complex intervention that aims at increased utilisation of primary and child health services. This unique collaborative effort includes key stakeholders from the Ethiopian health system, the implementing non-governmental organisations and universities, and combines state-of-the art effectiveness estimates and process evaluation with capacity building. The lessons learned from the project will inform efforts to engage communities and increase utilisation of care for children in other parts of Ethiopia and beyond.

**Trial registration:**

Current Controlled Trials ISRCTN12040912, retrospectively registered on 19 December, 2017.

## Background

Ethiopia reached the Millennium Development Goal 4, reflected in a reduction of the under-five mortality from 205 deaths per 1000 live births in 1990 to 64 in 2014 [1]. Neonatal mortality also decreased from 55 to 28 deaths per 1000 live births in the same period. The expansion of primary care services, improvements in nutrition [2], and progress across other sectors of society have reportedly contributed to reaching this goal. An analysis based on the Global Burden of Disease study reported neonatal conditions, together with lower respiratory tract infection and diarrhoeal diseases, as the dominant causes of under-five death [3].

As part of the Health Extension Program, in 2003 the Ethiopian Government introduced a new cadre of primary care workers called Health Extension Workers (HEWs) to reinforce efforts to improve maternal, newborn and child health in Ethiopia [4]. This category of health workers is reportedly able to correctly manage multiple child illnesses through the integrated community case management (iCCM) activities that the government initiated in 2010 [5]. If appropriately trained and supported, this cadre can also treat severe infections of the newborn in a cost-effective way within the Community-Based Newborn Care (CBNC) program that has been running since 2014 [6]. Although nearly all the HEWs throughout the country have been trained, relatively few sick newborns have been identified and treated [7]. Since 2011 a volunteer cadre called the Women’s Development Army (WDA-also known as the Health Development Army or Women’s Development Group) is active in promoting the use of health services [8, 9]. There are two levels to the WDA leaders. The smaller unit is comprised of 6 women, with one serving as a leader. Five or six of these networks are combined to form a group. The approximately 30 women in one group are led by one of the network leaders. The WDA leaders work closely with HEWs to promote maternal and child health services.

In Ethiopia, the utilisation of maternal health services shows disparities between regions and social groups [10, 11], and skilled birth attendance, although increasing, has remained inadequate with increasing social inequity [12]. Insufficient number of sick children are taken to HEWs for treatment of common childhood diseases, and the expansion of the iCCM program has not created sufficient community demand for use of these services [13].

The low utilisation of child health care calls for concerted efforts to improve the equitable reach of high-quality services [14, 15]. This background formed the rationale for developing a complex intervention to engage the community, strengthen the capacity of primary care workers to provide high-quality services and reinforce the local ownership and accountability of the primary level maternal and child health services. It was hypothesized that, given the low service utilization, increased awareness and promotion of primary health services in combination with strengthened provision of iCCM and CBNC and improved ownership and accountability of these services would lead to increased service use, which ultimately would further reduce neonatal and under-five mortality.

Thus, the evaluation described in this protocol aims to assess whether the proportion of children under the age of 5 years with suspected pneumonia, diarrhoea, fever or neonatal sepsis, that seek and receive appropriate care has increased in intervention as compared to comparison areas. Further, we present a plan for whether the information on the fidelity, reach, and dose of the interventions support the plausibility of changes in the outcome, and provide insights into the pathways of effects, feasibility, and program cost-effectiveness. Findings of this evaluation will be used to improve existing platforms of service delivery in the country. It is anticipated that successful interventions will be scaled up nationally provided sufficient budget support is available.

## Design and methods

### Setting

**Fig. 1:**
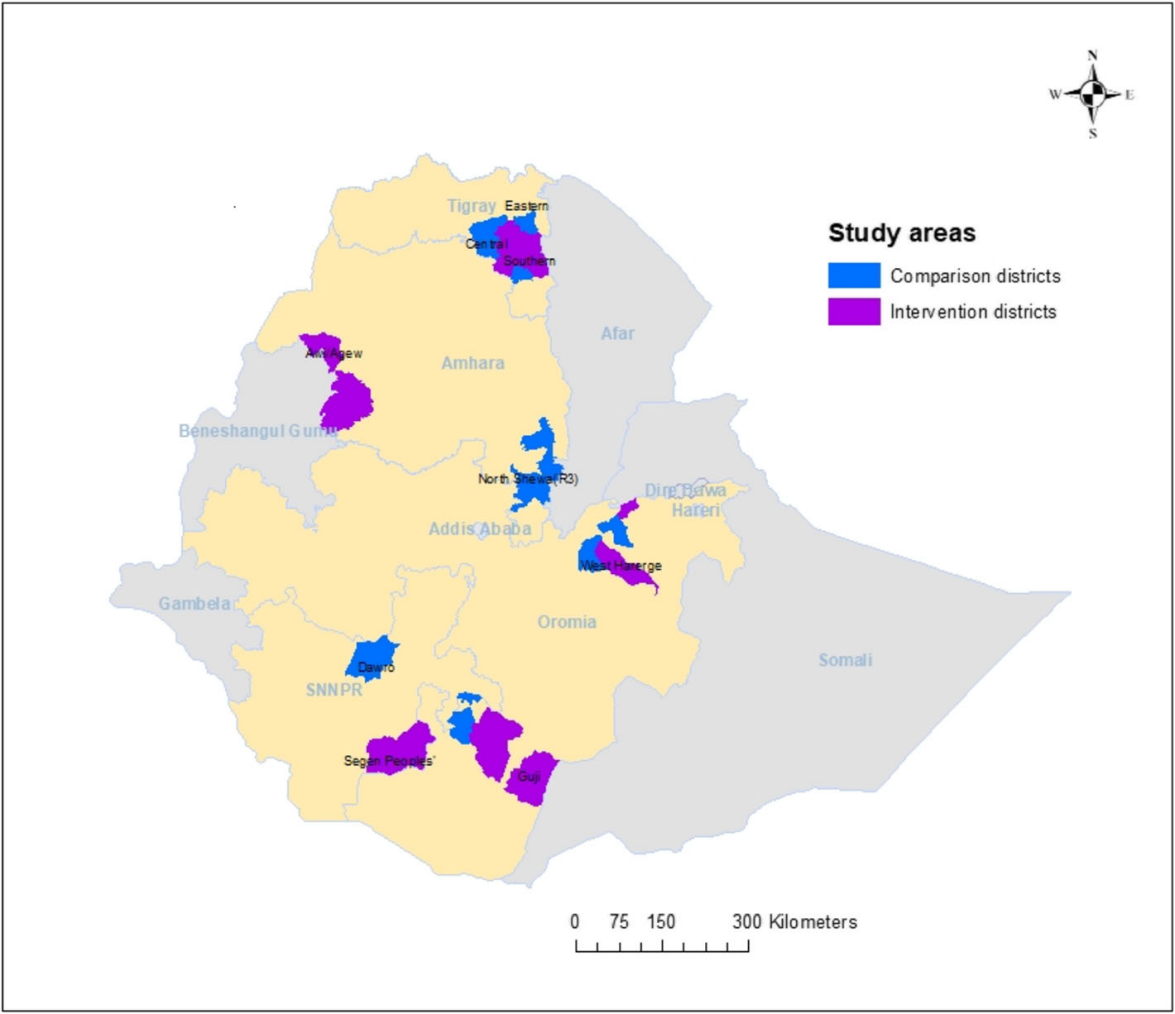
Invervention and comparsion areas for the evaluation of Optimzting the Halth Extention Program intevention, Ethiopia. Produced by the authors using ArcGIS 10

The Federal Ministry of Health in collaboration with the non-governmental partners UNICEF, Last 10 Kilometres/John Snow, Inc., Save the Children, and Program for Appropriate Technology in Health (PATH) initiated the “Optimizing the Health Extension Program” project to increase the utilisation of primary child health services. This evaluation has a pragmatic trial design with purposefully selected 26 intervention and 26 comparison districts (woredas), with a total population of 8 million, across four regions of Ethiopia (Amhara, Southern Nations Nationalities and People, Oromia, and Tigray). Figure 1 shows the intervention and comparison areas within these four regions.

The intervention areas were selected by government and implementing partners for having a relatively low utilization of primary child health services. The Regional Health Bureaus in these regions, with the support from their local universities, selected the comparison districts to match the intervention districts. Selection was based on demographic and health criteria that included population size, number of primary health care units, burden of diseases, health service performance data, length of time since iCCM and CBNC program initiation, prior exposure to other similar programs, and absence of non-governmental organizations addressing demand generation. The intervention, which started in 2016, has an intended duration of 2.5 years and is based on an analysis of barriers to the utilisation of newborn, child and maternal health services. The planned evaluation follows a plausibility approach [16]. It includes analysis of difference in differences of outcomes and a process evaluation of the intervention in line with the UK Medical Research Council’s guidelines [17].

The baseline and endline surveys, as well as the process evaluation are implemented by the London School of Hygiene & Tropical Medicine (LSHTM) and Ethiopian Public Health Institute (EPHI) along with representatives from Gondar, Jimma, Mekelle and Hawassa Universities. A steering committee comprising representatives from each of the universities, implementing partners, Ethiopian Public Health Institute and Federal Ministry of Health was established to meet quarterly. The committee provides advice on the evaluation of the project and assists in resolving issues encountered during the course of the evaluation. Given that the Optimising the Health Extension Program was a community and health system level intervention, a data monitoring committee was not deemed necessary.

### Barrier analysis

Key Ethiopian governmental and non-governmental stakeholders in the field of maternal, newborn and child health services met in 2016 for a facilitated workshop where the perceived demand- and supply-side barriers to CBNC and iCCM service utilisation were identified. The demand-side barriers included perceived lack of knowledge of diseases and danger signs [18], and lack of awareness of what primary level services could offer. Further, it was suggested that families often have a preference for traditional healers and home remedies [19] and that the higher availability of services offered by private providers was also appreciated in the households [20]. In a qualitative analysis of barriers to care-seeking for common childhood infectious diseases, the trust in the primary care services was low [21]. The barrier analysis showed a lack of community awareness of the curative, as well as preventive services provided by the HEW and that the quality of care on the primary level was perceived to be low [22]. Also, there was a felt need to strengthen the HEWs in supporting pregnant women in birth preparedness, referral to midwives, and institutional delivery [23]. All the above listed barriers resulted in under-use of maternal, newborn and child health services. Other demand side barriers included delay in seeking care due to the need to obtain husbands’ permission and financial support, the perceived cost and real cost of travel, particularly due to costs associated with referral of severely ill children to a further facility and repeated travel due to health post closure [21]. Supply-side barriers included frequent stock-outs of medicines and other necessary supplies, service interruption and inconsistent operating hours at health posts [24]. Further, it showed that HEWs had poor skills and confidence [25], especially when managing and treating newborns [26], lack of local government ownership and lack of accountability for both the CBNC and iCCM programs, and inconsistent supervision and monitoring. This barrier analysis formed the basis of a logic framework for a complex intervention that postulated that community engagement would increase care seeking for ill children, capacity building would improve availability of quality of CBNC and iCCM services and district level ownership and accountability would improve integration of these services into the district level planning and budgeting (Table 1). Together these three strategies would lead to an increased utilization of CBNC and iCCM services.

**Table 1:** Logic framework for the Optimizing Health Extension Program intervention in selected districts of Ethiopia

Assumptions	• Local stakeholders committed to coordinate and support the interventions • Traditional leaders will promote the maternal, newborn and child health services • The government health sector and supply chain partners will ensure drug and service availability		
Strategies	COMMUNITY ENGAGEMENT	CAPACITY BUILDING	OWNERSHIP, ACCOUNTABILITY
Interventions	• Health post open house • Group discussions led by Women’s Development Army (WDA) members • Reaching male partners • Engaging schools • Engaging religious and traditional leaders • Health films • Radio spots and dramas	• WDA level one training • Community-based data for decision making • Health Extension Worker (HEW) gap filling training and job aids • Supportive supervision of HEWs • Performance review and mentorship meetings with HEWs • Provision of job aids and tools	• Advocacy for the integration of Community-Based Newborn Care (CBNC) and integrated community case management (iCCM) into planning, budgeting, management, and information systems of the district and sub-district levels. • Management standard for health post opening hours • Ambulance service for children’s referral • Engage Kebele (sub-district) command post in the efforts Establish community feedback mechanism
Output	• Awareness of childhood illness and availability of CBNC and iCCM • Acceptance of health post care • Evidence-based social and behavioural change communication	• WDA members capacitated • HEWs gained skills • Supportive supervision and performance review and mentorship meetings with HEWs done	• CBNC and iCCM integrated in the planning, management and information systems at district and sub-district levels • Standard set for health post opening hours • Sub-district level local administration engaged in demand creation and support to primary health service provision • Community feedback mechanisms created • Advocacy to decision makers and influential bodies
Intermediate outcomes	• Improved child health practice at household and community levels Data source: Household module • Improved availability of high quality community-based newborn care and integrated community case management of childhood diseases Data source: Health post, health extension worker and health provider assessment module • Improved ownership and accountability of community-based newborn care and integrated management of childhood illnesses Data source: woreda contextual factors module		
Outcome	• Increased utilisation of good quality community-based newborn care and integrated management of childhood illnesses Data source: household module		
Impact	• Reduction of under-five mortality		

### The intervention and underlying assumptions

The package of interventions to be implemented across 26 districts includes three interlinked strategies with possible synergies: [1] community engagement activities that aim at increasing the awareness of newborn and child diseases, the recognition and acceptance of the care provided on the primary level, and the formulation of action plans at the local level [2]; capacity building of HEWs and WDA leaders such as gap filling training, supportive supervision and mentorship to improve iCCM and CBNC services, and [3] strengthening the local government’s ownership and accountability of the primary newborn and child health services by advocating for the sustained integration of CBNC and iCCM into the planning, budgeting, monitoring, management and support systems of the district and sub-district level (Table 1). Assumptions made by the implementers to achieve the success of the Optimizing the Health Extension Program included support from local stakeholders, traditional and religious leaders, governmental health sector and supply chain partners.

### Implementation

The Ethiopian Government, in collaboration with PATH and UNICEF (through sub-contractors Save the Children and Last 10 Kilometres) implement the intervention. These organizations have quarterly meetings to harmonize the intervention activities across the 26 intervention districts.

Trained professionals from the implementing organizations and the public sector facilitate the community engagement activities (Table 1). To raise community awareness of iCCM and CBNC services, implementers organise health post open house sessions to introduce available services and conduct workshops with schoolteachers and religious leaders. Community engagement will also be supported by behaviour change communication materials (brochures, posters, and banners). Educational films developed by implementers will be screened in health facilities and similarly radio messages and dramas will be developed and broadcast in implementation districts. Health professionals within the district health services facilitate the capacity building activities. For example, midwives from health centres provide training to HEWs, while HEWs provide training for WDA leaders. Where missing, implementers will provide registration books, iCCM and CBNC treatment algorithm booklets and backpacks to carry these items for community level service provision. Facilitators from the implementing partners lead the ownership and accountability activities. District ownership efforts include advocacy workshops at district and sub-district levels and support to the annual district health planning sessions.

The implementers plan to achieve a high reach of the different innovations across all intervention districts. Thus, the ambition is that the community meetings, school engagement activities, training of WDA leaders and HEWs, as well as district ownership efforts should reach all areas and relevant stakeholders.

### Process evaluation, including mechanisms of impact, and contextual factors

**Fig. 2:**
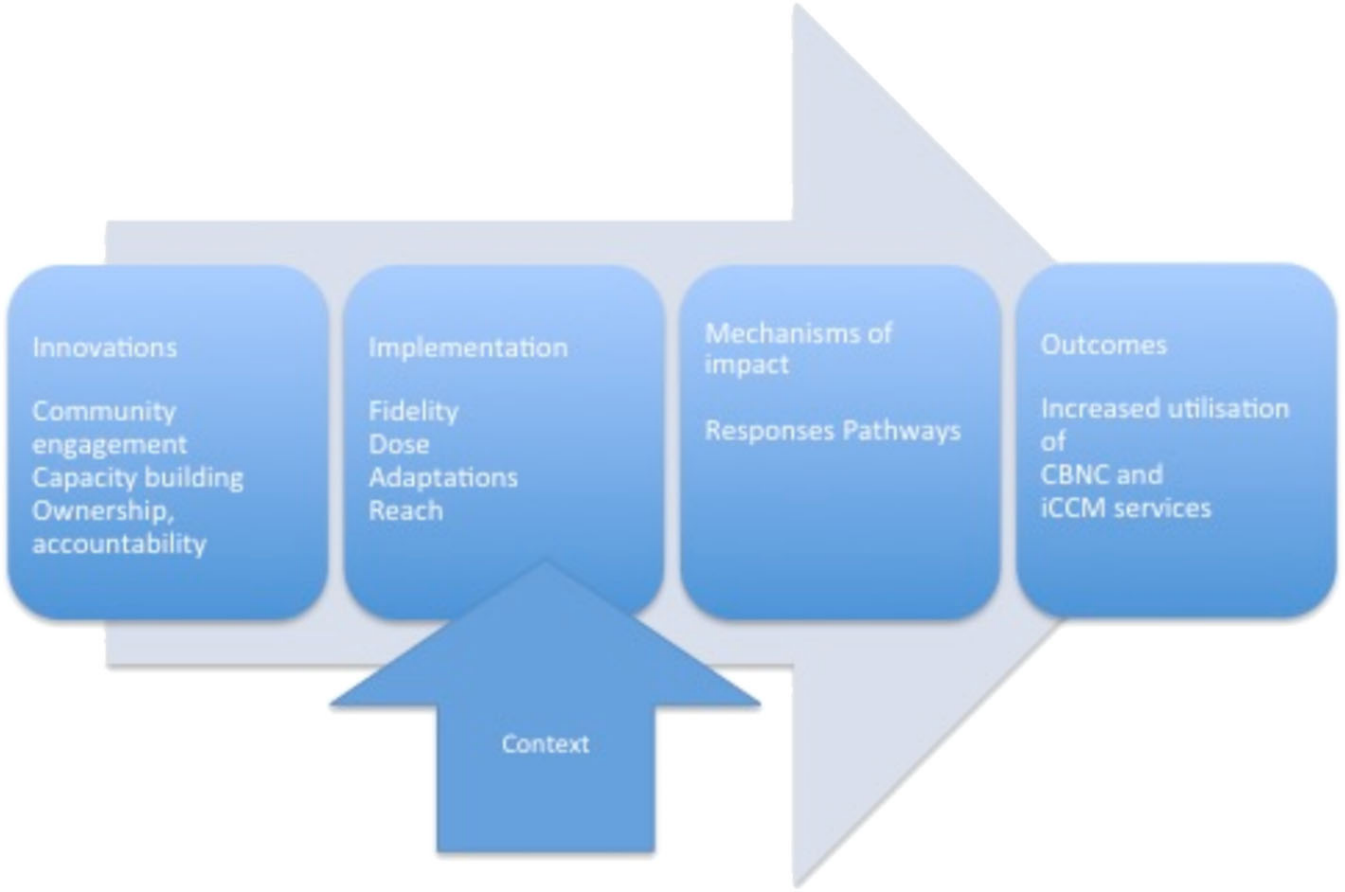
Framework for the process evaluation of the Optimizing the Health Extension Program intervention

The process evaluation is guided by the UK Medical Research Council framework for complex interventions [17]. A graphical representation of the process evaluation is provided in Fig. 2.

The implementing partners will prospectively collect data on the implementation of the interventions. The innovations within the three strategies will continually be harmonized across the implementing partners. Some variations between implementers and geographies was intended due to the different contexts of the districts. Such variations can be captured through the process evaluation. Implementers will also make efforts to harmonize the corresponding data sources. These databases will include information on each performed activity, including information on the innovation, the facilitator(s), the recipients, the health-system level and place, and timing of each activity. The implementing partners will also provide information on training and support to deliver the interventions and any changes made in some parts of the intervention districts or across all districts. This information will be aggregated to describe the fidelity (whether an intervention was delivered as intended), reach (how much of the intended audience was exposed to the intervention), and where feasible the dose (how much of the intervention was received by the intended audience) and adaptations (originally unintended changes made to an intervention across all or in selected parts of the study areas). In the endline survey, the participants (mothers of children below the age of 5 years, WDA leaders, HEWs, woreda health office representatives) will be asked about their contact with the interventions to measure reach. The baseline and endline surveys include questions on the interactions between the households and both WDA members and HEWs to measure dose (Table 2). Overall, the implementation process data will allow for analyses of whether the Optimizing the Health Extension Program innovations were implemented according to plans and associated with better awareness and acceptance of CBNC and iCCM services, provision of improved quality of services, and strengthened ownership and accountability of CBNC and iCCM services, respectively. In addition, a qualitative study will be conducted to explore views and experiences of program planners, managers and implementers, identifying what components worked well and what was not successful in the delivery of Optimizing the Health Extension Program interventions.

**Table 2:** Baseline and endline survey questionnaires for the Optimizing the Health Extension Program intervention evaluation

Questionnaire modules	Content
Household module ***N*** **= 6000**	• Location of household using global positioning system (GPS) coordinates • Members of household • Characteristics of the house and assets ^a^ • Women of reproductive age • Birth history • Use of maternal and perinatal health services • Knowledge of child diseases and danger signs • Care seeking and treatment for child illness • Preventive behaviour
Health post module ***N*** **= 200** Halth centre module^b^	• Location of health post and health centre using GPS coordinates • Facility-level preparedness to provide child health services • Data extracted from registers • Supportive supervision and mentorship from health centres to health posts
Health extension worker module ^c^	• Knowledge on newborn and child health care • Training, supervision, mentorship • Services provided to newborns and children
Health centre staff module ^d^	• Knowledge on newborn and child health care • Training, supervision • Services provided • Working conditions
Health provider assessment of the quality of care for a sick child module ***N*** **= 800**	• Observation and re-examination of Health Extension Workers’ assessment, classification, and treatment of sick children at health post
Women’s development army module ***N*** **= 200**	• Training • Knowledge • Activities in promoting maternal, newborn, and child health
Woreda contextual factors module ***N*** **= 52**	• Demography • Maternal, newborn and child health programs • District resources and infrastructure • Training and supervision activities, • Recent epidemics and natural disasters
Context Assessment for Community Health (COACH) module ^e^ ***N*** **= 200**	• Available resources, • Community engagement, • Monitoring services for action, • Sources of knowledge, • Commitment to work, • Work culture, • Leadership, • Informal payment

^a^ Asset ownership will be used to estimate relative socio-economic status, using an asset index based on principal components analysis

^b^ Some health posts are served by the same health centre hence the exact samples size can’t be determined

^c^ All the health extension workers in each health post will be interviewed. Due to the varying numbers of workers in health posts, the exact sample size can’t be determined

^d^ We will interview one staff per sampled health centre

^e^ Conducted at endline survey only with one HEW in each health post

### Effectiveness of the optimizing the health extension program

The baseline and endline surveys in intervention and comparison areas will include modules for household, facility preparedness to provide child health services, the health worker, a quality of care assessment, the WDA leader and woreda contextual factors. As part of the endline survey the HEWs will be interviewed, using the questionnaire-based Context Assessment for Community Health (COACH) tool [27]. This validated tool measures eight aspects of the context in which the HEWs work.

### Outcomes

*The primary outcome* is care seeking (at health posts, health centres, hospitals and clinics) for an illness in under-fives. *Secondary outcomes* include appropriate treatment for diarrhoeal diseases (oral rehydration therapy, zinc tablets), probable pneumonia (antibiotics), fever and malaria, and neonatal sepsis (antibiotics); improved knowledge towards childhood illnesses and treatment services among caregivers of under-5 children; improved attitudes or perceptions towards the iCCM and CBNC services at health posts; improved iCCM and CBNC program ownership by the public health sector (including inclusion of iCCM and CBNC indicators in their planning and budget allocation); and, improved availability of quality iCCM services provided by the HEWs.

*The sample size* for baseline and endline surveys is based on the requirement that these surveys should have adequate power to measure changes in a fixed number of percentage points between intervention and comparison areas from start to end of the study. For the household survey, the sample size was powered for the main outcome of care seeking for any illness in the 2 weeks prior to the survey (Table 3). Sampling 30 households in the 100 selected enumeration areas would yield 3000 households per group. The Ethiopia Demographic Heath Survey (DHS) data has shown that the rate of children under-five to households surveyed was 0.65. Based on this assumption, a sample survey of 3000 households per group would be expected to achieve a sample size of 1747 children below the age of 5 years. The evaluation of the integrated management of childhood illness in Tanzania reported 50% of under-five children to have had an illness in the 2 weeks prior to the survey [28]. We assumed a more conservative 30%. Based on the calculations of a sample size of 3000 households per group (6000 in total) with 90% completeness and a design effect of 1.3, we would have 80% power to detect differences of 10–20 percentage points across the range of child health indicators as statistically significant at the 5% level.

**Table 3:** Sample size for before vs. after comparison of sick child care seeking

Indicator	Expected level at baseline survey	Households per group required for a 10, 15 or 20 percentage point increase^a^		
Care seeking		10	15	20
% of children aged 2–59 months who were reported to have an illness in the past 2 weeks for whom advice or treatment was sought from an appropriate provider	55%	12,210	5299	2893

^a^Assuming 80% power, and using the baseline design effect of 1.001 and 94% completeness

For the survey to assess the quality care provided by HEWs, with the assumption that each of the sampled enumeration areas will be served by one health post, the survey will include 100 intervention and 100 comparison area health posts for the quality of care assessment, where in each health post, the HEWs’ assessment of four sick children mobilized to come to the health post will be observed, followed by a re-examination of the child by a health officer. This will yield a sample size of 400 children in each group. A total of 800 children, with a design effect of 1.4, will have 80% power to detect a minimum of 15 percentage point changes in the correct classification of iCCM illnesses between intervention and comparison area HEWs at baseline and endline as statistically significant at the 5% level (Table 4).

**Table 4:** Sample size for before vs. after comparison of sick children aged 2–59 months correctly managed

Indicator	Expected level at baseline	Children per group required for a 10, 15 or 20 percentage point increase^a^		
Children correctly managmed		10	15	20
% of sick children aged 2–59 months who were correctly managed	50%	892	391	216

^a^Assuming 80% power, design effect of 1.4, 90% completeness

*The questionnaire tools for baseline and endline surveys* were adapted from the team’s own, and others’ previous work on the Integrated Management of Childhood Illness [28], iCCM [29], and CBNC [30]. The questionnaire instrument included modules regarding household, health posts and health centres, HEWs, health centre staff, WDA members, an observation and re-examination of the HEW assessing sick children, and district contextual factors (Table 2) [see additional files 1–9].

*The baseline survey* took ten weeks and was completed in February 2017 in intervention and comparison areas to assess the situation before the intervention. A two-stage stratified cluster sampling was applied in these two surveys using lists of enumeration areas of the 52 intervention and comparison districts from the latest (2007) Ethiopian census as the sampling frame. In the first stage, a list of all enumeration areas of the study districts were based on the 2007 Ethiopian Housing and Population Census. Two hundred enumeration areas were selected from 52 districts with probability proportional to size. Each enumeration area formed one cluster, and these clusters constituted the primary sampling unit. In the second stage, a systematic random sampling technique was applied to select 30 households in each cluster. All women aged 13 to 49 years who lived in the selected households were included, in order to identify women who had a live birth in the 12 months prior to the survey to assess care seeking in the neonatal period. Furthermore, children under the age of 5 years were included to assess care seeking for any illness in the 2 weeks prior to the survey. For every cluster, the WDA leader serving the cluster was interviewed. The health post and the HEWs serving the selected cluster, the health post’s referral health centre, and staff, and the district health office providing support to the selected facility were approached with survey modules. All study participants were sampled without replacement. Up to three visits were made to each participant to maximize their inclusion into the study.

To evaluate the quality of assessment and care provided to sick children at the health posts, an observation of a sick child consultation with an HEW and a re-examination by a child health officer was performed. Given that very few sick children are brought to the health post each day, data collectors mobilized the community to bring sick children on the day of the survey to ensure the required sample size is met.

Data collection teams and supervisors were trained over the course of ten days. They were not provided information on whether a district was an intervention or comparison area. Data were collected on tablets and encrypted data were regularly sent from the field to the Ethiopian Public Health Institute’s central server. The data manager then decrypted data and rigorous quality checks were conducted with feedback to the field teams. Data cleaning involved checking for errors, completeness and consistency. The data manager also ensured that all standards for data security, curation and access were met.

*The endline survey* is being conducted 2 years after the baseline survey, following the same procedures.

*The effectiveness assessment* will be based on a plausibility design [16], analysing difference-in-differences of the primary and secondary outcomes [31]. Data on primary and secondary outcomes will be analysed from baseline and endline household, health post, and health centre surveys in intervention and comparison districts, with adjustment for the cluster sampling and relevant confounding factors. The assessment will use blinded analysis. The code identifying the intervention and comparison areas will be revealed after the analysis and interpretations are completed.

Ten Ph.D. students from four Ethiopian universities, including candidates from the Regional Health Bureaus in the study provinces, and from the Ethiopian Public Health Institute, are engaged in the evaluation. PhD students were involved in the conduct of the surveys, participating in the training of data collectors and serving as regional survey coordinators. They have chosen topics for in-depth sub-studies linked to the evaluation, including equity in the utilisation of services; spatial analyses of care utilisation; quality of care provided by the HEWs; the role of WDA leaders in promoting the use of services; newborn care practices; the referral of sick children; and focused studies of care utilisation for diseases of the newborn and diarrhoeal diseases. Most of the students plan to use baseline and endline surveys for quantitative data and to perform qualitative studies within their chosen topics.

Findings from the effectiveness study, process evaluation and PhD research will be published as scientific articles and reports. No professional writers will be used. Publications arising from this evaluation will follow the recommendations from the International Committee of Medical Journal Editors [32].

### Trial status

The interventions were running up to the end of 2018 and endline survey started immediately thereafter. Data for process evaluation collection and analysis will take place until September 2019. Data analysis for the effectiveness study will be performed from mid of 2019 onwards.

## Discussion

This protocol describes an evaluation of a complex intervention that aims at an increased utilisation of primary child and maternal health services in Ethiopia. The interventions are based on a logic framework developed from an analysis of barriers to the utilisation of primary maternal and child health services. The program includes innovative components to engage the community, strengthen the capacity of primary care workers, and reinforce the local ownership and accountability of the primary maternal and child health services. This evaluation combines state-of-the-art effectiveness and process evaluation with capacity building and training of Ethiopian Ph.D. students from universities in the four study regions of Ethiopia, and is linked to regional health bureaus. We will assess the effectiveness of the intervention to increase the seeking of appropriate care by difference-in-differences analysis based on baseline and endline surveys. The process evaluation follows the guidelines of the UK Medical Research Council.

### Barriers to health care utilisation

An analysis of barriers to the utilisation of child and maternal health primary services was the point of departure for the development of a logic framework of the interventions. Representatives of the Ethiopian health system and non-governmental organizations active in maternal, newborn and child health reviewed findings form a qualitative barrier analysis study carried out by local researchers and a literature review conducted by UNICEF. The findings were in line with recently published literature from Ethiopia that had addressed these questions.

### Outcomes

The primary outcome of this intervention is care seeking for the major causes of death in children below the age of 5 years in Ethiopia: diarrhoeal diseases, pneumonia, and malaria [3]. Care seeking for suspected pneumonia was especially low in Ethiopia in a study comparing different countries in sub-Saharan Africa [33]. When measurements are challenging, as is the case for assessing antibiotic treatment for children with pneumonia, we will ensure findings are interpreted with caution [34]. A study in Eastern Ethiopia stressed the importance of having previous knowledge and experience of rehydration therapy and an established contact with the health post to seek care when the child suffers from diarrhoea [35].

### Three intervention strategies

The complex intervention includes innovations and activities to engage the community, build the capacity of the HEWs and leaders of the WDA, and foster ownership and accountability of the CBNC and iCCM programs by local health authorities.

Different community engagement interventions are increasingly getting attention for maternal, newborn, and child health. The evidence is mounting that such interventions may be effective to improve utilisation of services and get improved health outcomes [36]. Ethiopian studies showed that community engagement activities could expand the use and quality of perinatal care [37] and increase the coverage of postnatal care [38]. Community support to iCCM activities and community health workers is needed for sustained health benefits coupled with a focus on strengthening and enabling the public health system [39].

Although demand-side interventions may increase utilisation, this does not necessarily lead to better child and maternal outcomes, for which good quality, timely care is essential [40]. Training of primary health workers in CBNC and iCCM programs is a prerequisite, and Ethiopian experiences indicate that the knowledge gained may be satisfactorily retained, although refresher training is advisable for sustained effect [25, 41]. Supportive supervision and performance review meetings may be effective strategies to improve the quality of services provided by HEWs [42]. A review paper from several low-income countries reported that high-quality supervision focusing on supportive approaches, community monitoring, and problem-solving may be an effective solution [43].

In a review of data from facilities in eight mainly sub-Saharan African countries, the facility infrastructure was poorly associated with the observed quality of maternal, newborn, and child health care provided [44]. We have such infrastructure data from baseline and endline surveys that are essential to describe the setting, although it may not shed light on the intervention’s effect on the quality of services provided by the HEWs. In a meta-review of almost 100 systematic reviews of interventions to improve the quality of care, the facilitators and barriers were in the domains of information, patient-population engagement, leadership, regulations and standards, organizational capacity, models of care, communication, and satisfaction [45]. The COACH questionnaire tool that is planned to be part of the endline assessment with HEWs covers such domains and may potentially provide explanations as to whether the interventions have improved the quality of care provided or not [27]. Data on the inner as well as the organizational context offer opportunities to analyse the potential influence of these factors on the process and outcomes of the intervention.

As described above, the CBNC and iCCM programs implemented within the Ethiopian primary care context have proven to be effective in reducing mortality. For further quality improvements and sustained effects, the ownership and accountability for these programs on all levels of the health system must be considered [46]. Efforts to increase accountability for maternal and child health services might be more successful if many stakeholders get involved [47].

While the interventions rolled out in Ethiopia will be tailored to address the critical bottlenecks identified and therefore may be country-specific, the approaches to address some of the demand and service challenges may apply to other countries. As more countries begin to adopt iCCM, CBNC, and other community-based services to extend child and maternal health care beyond health facilities, lessons learned from Ethiopia may prove useful for other settings. Lessons learned from this evaluation can serve to adopt approaches in countries already rolling out iCCM and CBNC or feed into policy and implementation strategies for new countries planning to develop community-based platforms of service delivery.

## Conclusion

This protocol describes the evaluation of a complex intervention in four regions of Ethiopia that aims at increasing utilisation of primary care services for mothers and children. The intervention is based on an analysis of barriers to the utilisation of services and includes innovations to engage communities, train and support primary care workers, and promote ownership and accountability of CBNC and iCCM programs. Representatives of the health system, non-governmental organisations, and universities work together to learn, evaluate, and strengthen university capacity for health systems and implementation research, which will enhance sustainable work relationship between universities and Regional Health Bureaus. The process and outcome evaluations will inform the possible scale-up of efforts to increase primary health care utilisation for sick children in Ethiopia and similar settings.

## Data Availability

A data sharing committee has been established comprised of each of the universities and Ethiopian Public Health Institute. Any requests will be reviewed by this committee and if requests are granted data will be shared without any identifiers. Request for data can be made to Yemisrach B. Okwaraji (Yemisrach.Okwaraji@lshtm.ac.uk) or Desta Wolassa (desta.wolassa@lshtm.ac.uk).

## Abbreviations

CBNC: Community-Based Newborn Care
COACH: Context Assessment for Community Health
DHS: Demographic Heath Survey
EPHI: Ethiopian Public Health Institure
HEW: Health Extension Worker
iCCM: Integrated Community Case Management
LSHTM: London School of Hygiene & Tropical Medicine
PATH: Program for Appropriate Technology in Health
WDA: Women’s Development Army
